# Integrating Artificial Intelligence and Biotechnology to Enhance Cold Stress Resilience in Legumes

**DOI:** 10.3390/plants14172784

**Published:** 2025-09-05

**Authors:** Kai Wang, Lei Xia, Xuetong Yang, Chang Du, Tong Tang, Zheng Yang, Shijie Ma, Xinjian Wan, Feng Guan, Bo Shi, Yuanyuan Xie, Jingyun Zhang

**Affiliations:** 1 Institute of Vegetables and Flowers, Jiangxi Academy of Agricultural Sciences, Nanchang 330200, China; wangkai@jxaas.cn (K.W.); xialeijx@163.com (L.X.); yangxuetong2023@163.com (X.Y.); xinjian71@163.com (X.W.); guanfeng_0813@163.com (F.G.); shibo_jiangxi@163.com (B.S.); m18073933758@163.com (Y.X.); 2 Jiangxi Key Laboratory of Horticultural Crops (Fruit, Vegetable & Tea) Breeding, Jiangxi Academy of Agricultural Sciences, Nanchang 330200, China; 3 Jiangxi Engineering Research Center of Vegetable Molecular Breeding, Jiangxi Academy of Agricultural Sciences, Nanchang 330200, China; 4 Guangdong Provincial Key Laboratory of Biotechnology for Plant Development, School of Life Science, South China Normal University, Guangzhou 510631, China; duchang@m.scnu.edu.cn; 5 Department of Computer Science and Information Technologies, Elviña Campus, University of A Coruña, 15001 A Coruña, Spain; tongtang@scu.edu.cn; 6 Zhengzhou Research Base, State Key Laboratory of Cotton Bio-Breeding and Integrated Utilization, School of Agricultural Sciences, Zhengzhou University, Zhengzhou 450001, China; yangz@zzu.edu.cn; 7 Crop Research Institute, Anhui Academy of Agricultural Sciences, Hefei 230031, China; mashijieaaas@163.com

**Keywords:** cold stress, artificial intelligence, machine learning, advanced sensor technologies, legume

## Abstract

Cold stress severely limits legume productivity, threatening global food security, particularly in climate-vulnerable regions. This review synthesizes advances in understanding and enhancing cold tolerance in key legumes (chickpea, soybean, lentil, and cowpea), addressing three core questions: (1) molecular/physiological foundations of cold tolerance; (2) how emerging technologies accelerate stress dissection and breeding; and (3) integration strategies and deployment challenges. Legume cold tolerance involves conserved pathways (e.g., *ICE-CBF-COR,* Inducer of CBF Expression, C-repeat Binding Factor, Cold-Responsive genes) and species-specific mechanisms like soybean’s *GmTCF1a*-mediated pathway. Multi-omics have identified critical genes (e.g., *CaDREB1E* in chickpea, *NFR5* in pea) underlying adaptive traits (membrane stabilization, osmolyte accumulation) that reduce yield losses by 30–50% in tolerant genotypes. Technologically, AI and high-throughput phenotyping achieve >95% accuracy in early cold detection (3–7 days pre-symptoms) via hyperspectral/thermal imaging; deep learning (e.g., CNN-LSTM hybrids) improves trait prediction by 23% over linear models. Genomic selection cuts breeding cycles by 30–50% (to 3–5 years) using GEBVs (Genomic estimated breeding values) from hundreds of thousands of SNPs (Single-nucleotide polymorphisms). Advanced sensors (LIG-based, LoRaWAN) enable real-time monitoring (±0.1 °C precision, <30 s response), supporting precision irrigation that saves 15–40% water while maintaining yields. Key barriers include multi-omics data standardization and cost constraints in resource-limited regions. Integrating molecular insights with AI-driven phenomics and multi-omics is revolutionizing cold-tolerance breeding, accelerating climate-resilient variety development, and offering a blueprint for sustainable agricultural adaptation.

## 1. Introduction

Leguminous crops are fundamental to global food security, contributing significantly to world crop production and serving as essential protein sources for billions of people worldwide [1,2]. The Fabaceae family, which includes economically important species such as chickpea (*Cicer arietinum*), soybean (*Glycine max*), field pea (*Pisum sativum*), cowpea (*Vigna unguiculata*), and lentil (*Lens culinaris*), faces mounting challenges from climate variability and temperature extremes [3,4].

Cold stress significantly restricts the geographical distribution and productivity of these crops, causing yield losses of up to 60–70% in susceptible genotypes under severe conditions [5,6]. In recent years, cold stress alone has led to significant declines in the overall productivity of legume crops, with reductions of approximately 60% in chickpea and soybean, and around 70% in mungbean. Compared to unaffected plants, cold stress results in an average 24% reduction in soybean yields [7,8]. The economic impact is substantial, with global legume production losses due to various abiotic stresses, including cold stress, affecting food security, particularly in developing countries where legumes serve as primary protein sources [9,10]. Climate change projections indicate increasing temperature variability, including more frequent and unexpected cold snaps during critical growth stages. This climatic uncertainty threatens the expansion of legume cultivation into higher latitudes and altitudes, complicating efforts to meet the projected global food demand for 9.7 billion people by 2050 [11,12]. The reproductive phase of legumes is especially vulnerable to cold stress. Exposure to temperatures below 10 °C during flowering can cause pollen sterility and pod abortion, leading to complete yield loss under extreme conditions [13,14]. Moreover, the narrow genetic base of many domesticated legume species, a consequence of historical breeding bottlenecks, limits the natural variation available for cold-tolerance traits [15,16]. Traditional approaches to improving cold tolerance in legumes have relied heavily on phenotypic evaluation and conventional breeding, often requiring 8–12 years to develop new varieties through recurrent selection [17,18]. The complex, quantitative nature of cold tolerance, which is regulated by multiple genes and intricate networks controlling cellular membrane stability, osmotic adjustment, and antioxidant defense, demands more sophisticated analytical methods [19,20]. Conventional breeding also faces challenges in accurately phenotyping cold tolerance due to environmental variability, genotype-environment interactions, and the subjective nature of visual scoring [21,22]. The advent of artificial intelligence (AI), advanced sensor technologies, and big data analytics presents unprecedented opportunities to accelerate the discovery and deployment of cold-tolerant legume varieties. These converging technologies enable the integration of molecular, physiological, and environmental data streams, offering comprehensive insights into cold stress responses with unprecedented temporal and spatial resolution [23,24]. Machine learning applied to large-scale genomic and phenomic datasets has uncovered previously unrecognized patterns in stress response mechanisms, facilitating the identification of novel targets for genetic improvement [25,26]. Recent advances have led to the development of high-throughput phenotyping (HTP) platforms capable of processing thousands of plants rapidly and precisely, generating terabytes of multidimensional data per growing season [27,28]. Combining RGB imaging, hyperspectral sensors, thermal cameras, and 3D reconstruction technologies, these platforms detect stress responses days before visible symptoms emerge, enabling early intervention and more accurate trait assessment [24,29]. Deep learning algorithms, particularly convolutional neural networks, achieve over 95% accuracy in automated stress classification, surpassing human expert consistency and throughput [30,31]. Simultaneously, genomic selection approaches leveraging genome-wide association studies (GWAS) and multi-omics integration have improved prediction accuracies for complex traits while reducing breeding cycles from decades to years [32,33]. The integration of Internet of Things (IoT) sensors, precision agriculture technologies, and cloud-based analytics has revolutionized field-scale monitoring and management of legumes under cold stress [34,35]. Wireless sensor networks deployed across agricultural landscapes provide real-time environmental monitoring with subdegree precision, enabling automated irrigation, heating, and protective covering systems responsive to frost predictions [36,37]. Edge computing platforms process sensor data locally, reducing latency and enabling immediate responses while maintaining connectivity to cloud-based machine learning models for long-term trend analysis [38,39]. While existing reviews address cold stress in plants or agricultural technologies, they rarely focus specifically on legumes or integrate multiple disciplines. Most compilations either group legumes with other crops, overlooking their unique traits like nitrogen-fixing nodules and symbiotic relationships, or emphasize single technologies (e.g., genomic selection alone or sensor applications in isolation). Few synthesize the tripartite synergy of AI, sensor technologies, and genomics, particularly for resource-limited regions where legumes serve as primary protein sources. This review fills these gaps by focusing exclusively on legumes, integrating three key technological pillars, and emphasizing practical adaptations for low-input agricultural systems.

Integrate transcriptomic, proteomic, and metabolomic data with high-density Single-nucleotide polymorphism (SNP)arrays. This enables genome-wide prediction models. These models capture additive and epistatic effects. Such effects underlie cold-tolerance phenotypes [24,40]. Population genomics has identified adaptive introgression events and structural variants linked to cold adaptation, offering new breeding targets [4,41].

The reference of this review was screened through the PRISMA (Preferred Reporting Items for Systematic Reviews and Meta-Analyses) framework [42]. Firstly, systematically search the electronic database PubMed using a combination of keywords related to legumes, cold stress, AI, sensor technology, and multi-omics “((((((ai Legumes) OR (AI Sensor Technologies Legumes)) OR (AI multi-omics Legumes)) OR (AI GWAS Legumes)) OR (AI crisp legumes)) OR (AI cold stress Legumes))” in the PubMed database; 2555 records were screened in the last 20 years. After removing duplicates via reference management software EndNote, titles and abstracts will be screened against predefined inclusion criteria (e.g., integration of at least one of the three technologies) and exclusion criteria. Two independent reviewers will conduct this step, with discrepancies resolved through discussion, and 1158 records were screened. Then, full texts of the remaining studies will be assessed to confirm alignment with the review’s scope, ensuring they contribute meaningful insights into the integration of AI, sensor technology, and genomics for legume cold stress resilience. The final set of included studies will be subjected to data extraction and quality assessment.

**Figure 1:**
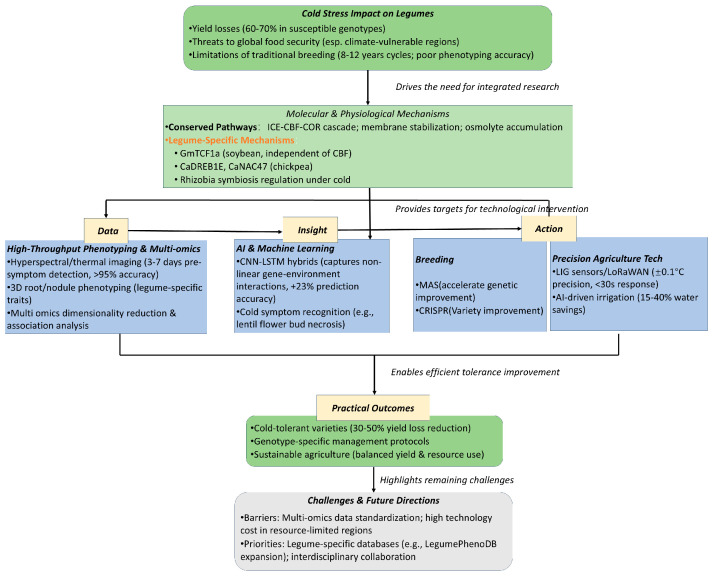
Schematic diagram of the manuscript’s reasoning.

This review addresses three core research questions: (1) What are the legume-specific molecular and physiological foundations of cold tolerance, distinct from other crops? (2) How can integrated technologies (AI, HTP, genomic selection, IoT) overcome traditional breeding limitations for cold tolerance in legumes? (3) What strategies and challenges enable translating these innovations into practical agricultural systems? Our objectives are to synthesize legume-specific cold-tolerance mechanisms, evaluate technological synergies accelerating breeding, and propose a roadmap for lab-to-field translation. This work makes three unique contributions: (1) a legume-centric focus highlighting species-specific traits like nodule cold responses; (2) cross-disciplinary integration linking molecular pathways (e.g., *ICE-CBF-COR* and legume-specific *GmTCF1a* [22]) with AI-driven phenomics and genomic selection; and (3) practical guidance on deploying innovations in resource-limited regions, where legumes are dietary staples. Unlike existing reviews that focus on single or two combined technologies (e.g., genomics and AI of legume stress) or non-legume crops, this is the first to systematically synthesize the tripartite integration of AI, sensors, and genomics specifically for legumes. By mapping technological synergies to breeding workflows, the review provides actionable tools for accelerating cold-tolerant legume development [43,44]. It highlights understudied areas (e.g., AI models for minor legumes, low-cost sensor deployment in resource-poor regions) that have been overlooked in fragmented existing literature. Subsequent sections build on this framework: Section 2 explores molecular and physiological mechanisms; Section 3, Section 4, Section 5, Section 6 analyze technological advancements (AI, sensors, genomics) and their integration; and Section 7 and Section 8 address implementation strategies and future directions. Together, these sections provide a cohesive narrative advancing legume cold-tolerance research through interdisciplinary integration (Figure 1).

## 2. Molecular Foundations of Cold Tolerance in Leguminous Plants

### 2.1. Core Regulatory Pathways and Mechanisms

**Figure 2:**
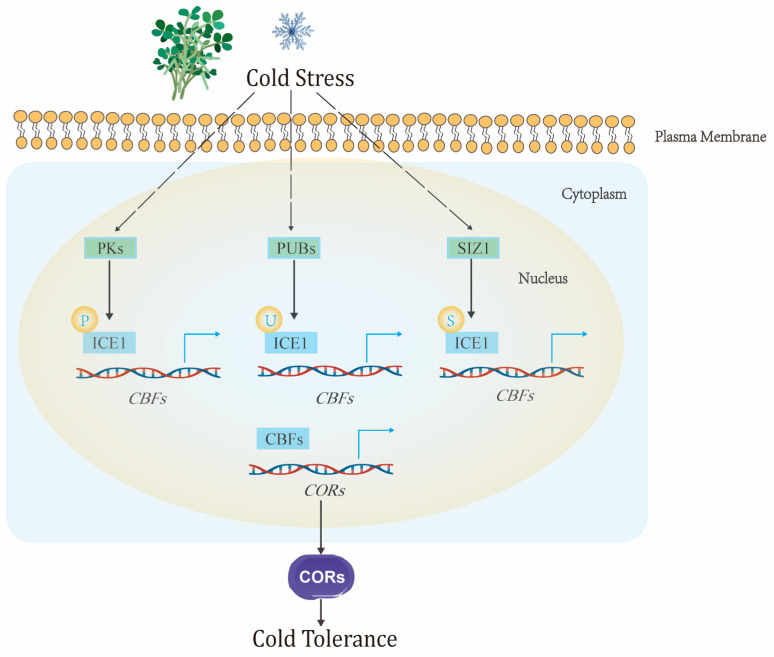
*ICE-CBF-COR* Signaling Pathway in Cold Stress Response. *ICE1-CBFs-CORs* were the key pathway in response to cold stress in plants. The plants could induce post-translational modifications (phosphorylation, ubiquitination, and SUMOylation) of ICE1 indirectly by perceiving low temperature. Then, ICE1 directly promotes the expression of *CBFs*; the expression of *CORs* was significantly induced by CBFs through binding to the promoter of COR; and finally, *CORs* improved the low-temperature tolerance of plants.

The molecular architecture of cold tolerance in leguminous plants centers on sophisticated multi-layered defense mechanisms operating at transcriptional, post-translational, and metabolic levels. The *ICE-CBF-COR* (Inducer of CBF Expression, C-repeat Binding Factor, Cold-Responsive genes) signaling cascade serves as the master regulatory hub across species [45,46] (Figure 2). This pathway initiates at the plasma membrane, where cold sensors perceive temperature changes and trigger downstream signaling events [47,48]. Recent transcriptomic analyses in chickpea have revealed that cold-tolerant genotypes demonstrate significantly different molecular responses compared to sensitive varieties, with 3710 differentially expressed genes identified in tolerant lines versus 3473 in sensitive genotypes [49]. Key transcription factors involved in cold tolerance include *CaDREB1E*, *CaMYB4*, *CaNAC47*, *CaTCP4*, and *CaWRKY33*, which regulate extensive downstream gene networks [49,50]. In soybean, the identification of *GmTCF1a* as a cold-specific regulator operating independently of the CBF pathway represents a significant breakthrough in understanding species-specific adaptations. This RCC1 family gene enhances freezing tolerance through mechanisms distinct from traditional cold response pathways, demonstrating the complexity of legume cold-tolerance mechanisms [51]. When ectopically overexpressed in Arabidopsis, *GmTCF1a* increased plant survival rates under freezing stress, reduced electrolyte leakage, and stabilized cellular structures [51]. Post-translational modifications provide rapid response mechanisms through phosphorylation cascades involving MAPK pathways, ubiquitination processes, and SUMOylation mechanisms that regulate transcription factor stability at low temperatures. These modifications enable dynamic control of cold response activation and duration, representing critical targets for biotechnological interventions [47,52,53]. Legumes exhibit unique cold response mechanisms tied to their biological features. Symbiotic nitrogen fixation (SNF), a critical process for legume nutrient acquisition, is particularly susceptible to low-temperature stress. Under cold conditions, SNF efficiency can plummet by up to 45%, triggering significant reductions in plant productivity due to impaired nitrogen supply. This species-specific trait highlights the need for legume-focused analyses absent from broader plant stress reviews.

### 2.2. Genomic Architecture and QTL Mapping

Recent genomic studies have identified multiple QTLs (quantitative trait loci) for cold tolerance in soybean through systems biology approaches, with individual QTLs explaining varying percentages of phenotypic variation. Multi-environment QTL analysis has revealed that several QTLs are consistently detected across germination and seedling stages, indicating opportunities for developing varieties with broad-spectrum cold tolerance [54,55]. The integration of whole genome sequencing with GWAS approaches has accelerated candidate gene identification. In chickpea, genome-wide transcriptional profiling identified major transcription factors and protective genes specifically associated with cold tolerance, including CaCDPK4 (Calcium-dependent protein kinases (*CDPKs*) are key mediators of Ca^2+^ signaling, involved in various biological processes such as parasite egress from host cells, outer membrane motility, invasion, and cell division) and *CaPP2C6* (Under well-watered conditions, osmotic signaling is inhibited primarily by the action of type 2C protein phosphatases (*PP2Cs*). These *PP2Cs* are evolutionarily conserved negative regulators involved in osmoregulation and function in relation to *SnRK2s. CaMKK2* mitogen-activated protein kinases (*MAPKs*) are crucial in various cellular processes, including stress responses, among others. Their activation occurs via a canonical cascade, which involves phosphorylation by upstream dual-specificity MAPK kinases (MKKs), and *CaHSFA3* as a key regulatory component. These findings provide molecular targets for marker-assisted selection and genetic engineering approaches to enhance cold tolerance in chickpea breeding programs [49,56].

### 2.3. Physiological Adaptations and Cellular Protection

Cold tolerance involves sophisticated membrane stabilization processes through increased fatty acid desaturation and enhanced proportions of unsaturated fatty acids. Tolerant genotypes show reduced electrolyte leakage compared to sensitive varieties, with membrane stability serving as a critical physiological marker [20,57]. Osmotic adjustment occurs through the accumulation of compatible solutes including proline, soluble sugars, glycine betaine, and trehalose, providing cellular protection during freezing stress [58,59]. Antioxidant defense systems demonstrate remarkable upregulation under cold stress, with superoxide dismutase, catalase, and peroxidase activities showing significant increases in tolerant varieties. These enzymatic responses combine with non-enzymatic antioxidants to maintain cellular redox balance and prevent oxidative damage during cold exposure [60,61].

## 3. AI Applications in Cold-Tolerance Research

### 3.1. Computer Vision and Deep Learning for Advanced Plant Phenotyping

The integration of computer vision and deep learning has revolutionized plant stress phenotyping, enabling automated, high-throughput, and precise detection of cold stress responses in legumes. Computer vision tools called Convolutional Neural Networks (CNNs) are transforming how we detect cold stress in legumes. Think of CNNs as ‘digital plant doctors’—they analyze images of leaves, stems, or canopies to spot early signs of stress, much like a farmer inspecting crops, but with far greater speed and precision. Three common types of CNNs are particularly useful here: ResNet acts like a ‘detail-focused inspector’ that can identify tiny changes, such as faint leaf yellowing or cell damage, even in complex plant structures, and it achieves 96–97% accuracy in spotting these early stress signs; VGG works best for detecting small, specific changes, like how cold alters the density of tiny pores (stomata) on leaves; EfficientNet balances speed and accuracy, making it practical for real-time field use—like a quick-check tool that farmers could use with mobile devices. These tools ‘learn’ from thousands of plant images, allowing them to spot stress 3–7 days before humans can see visible damage [62,63]. In plant leaf classification, four CNN models, Vision Transformer, ResNet-50, DenseNet-201, and Xception were tested; the accuracy rates are 99.75%, 98.28%, 99.51%, and 97.04% on the Swedish Leaf dataset. Based on the KNN model, the accuracy for the identification of the plant type is 96.1%. The CNN models VGG16 and VGG19 presented the accuracy of 95.62% and 96.01% in the detection of rice plant diseases [64,65,66]. However, their depth demands significant computational resources, limiting real-time field deployment. VGG architectures, characterized by stacked 3 × 3 convolutional layers (e.g., VGG-16), prioritize fine-grained feature extraction, making them ideal for detecting microstructural changes like stomatal density shifts under cold stress. Their simplicity aids adaptability to diverse legume species, but their large parameter count (e.g., ~138 million in VGG-16) reduces efficiency for large-scale phenotyping. EfficientNet addresses this trade-off through compound scaling, balancing depth, width, and resolution, delivering high accuracy with fewer parameters (e.g., EfficientNet-B4 has ~19 million). This efficiency enables real-time deployment on resource-limited field devices, critical for continuous cold stress monitoring [67], though it requires careful tuning and may miss ultra-subtle traits compared to ResNet. These three architectures dominate phenotyping because they collectively address core needs: ResNet captures complex, multi-scale stress traits; VGG extracts detailed microfeatures; EfficientNet balances speed and precision. Beyond CNNs, traditional machine learning (ML) methods, such as SVM and Random Forest, remain relevant for small datasets, though they rely on preprocessing steps like denoising (Gaussian filtering), segmentation (Otsu’s method), and handcrafted feature extraction (e.g., color, texture, and shape metrics). AI-driven segmentation techniques further enhance phenotyping: U-Net, with its encoder-decoder structure, excels at segmenting overlapping legume organs (e.g., tangled leaves); DeepLab uses atrous convolution to map field-scale stress spread; Mask R-CNN enables instance segmentation of individual plants, tracking stress progression. Recent advances have also yielded lightweight, plant-specific CNNs [24,68], optimizing computational efficiency for field deployment without sacrificing accuracy, further advancing high-throughput cold stress detection in legumes. An approach was created for segmenting and counting rapeseed pods precisely based on the Mask R-CNN model; with the precision rates about 90%, this model is helpful to identify and screen pods in leguminous plants, such as soybean, bean, cowpea, pea, and mung bean [69]. However, the work of CNN depends on large-scale annotated data and powerful computing resources; it is necessary to establish a phenotype database for leguminous plants. The comparison of AI and biotechnology for plant stress detection is described in Table 1.

### 3.2. Advanced High-Throughput Phenotyping Platforms

Technically and scientifically, High-Throughput Phenotyping (HTP) refers to an integrated technological system that employs automated sensing, imaging, and data processing workflows to quantify phenotypic traits of plants (morphological, physiological, biochemical, or developmental) at scale, with high temporal and spatial resolution, while minimizing manual intervention. It bridges plant genetics, agronomy, and data science by enabling the systematic acquisition of phenotypic data that correlates with genotypic information, facilitating the identification of genotype-phenotype associations critical for crop improvement and functional genomics. The term “high-throughput” denotes the capacity to process a large number of plant specimens or experimental units within a short timeframe, exceeding the throughput of traditional manual phenotyping. While specific thresholds vary by application (e.g., field vs. controlled environments), industry benchmarks typically define high throughput as in controlled environments (growth chambers, greenhouses), phenotyping ≥500 individual plants per hour, with automated handling of trays or pots; in field settings, scanning ≥10,000 plants per day using ground-based or aerial systems, covering hectare-scale plots [70]. These thresholds are contingent on the complexity of traits measured; for example, platforms focusing on simple morphometric traits (e.g., plant height) may achieve higher throughput than those quantifying hyperspectral biochemical signatures. Established HTP system brands and models, which have become industry standards, include LemnaTec Group (LemnaTec GmbH, Aachen, Germany), which offers modular systems such as the LemnaTec Scanalyzer 3D (greenhouse-based, integrating RGB, hyperspectral, and fluorescence imaging) (LemnaTec GmbH, Aachen, Germany) and LemnaTec Field Scanalyzer (field-deployed, with high-resolution LiDAR and multispectral sensors) (LemnaTec GmbH, Aachen, Germany), capable of processing 1000+ plants/hour in controlled environments; Phenospex with the Phenospex FieldScan series that combines RGB and multispectral imaging for field phenotyping, with a throughput of 15,000–20,000 plants/day; PlantEye (Phenosys) specializing in 3D plant reconstruction, where the PlantEye F500 (Phenospex, Heerlen, The Netherlands) achieves sub-millimeter resolution for canopy architecture traits, processing 600+ plants/hour in greenhouses; and aerial platforms like the DJI Matrice 300 RTK (Dji, Shenzhen, China) integrated with MicaSense Altum (multispectral + thermal) or Sentera 6X cameras (Sentera, Saint Paul, MN, USA), enabling field-scale phenotyping of 50+ hectares/day. Modern HTP platforms integrate diverse sensor modalities, including RGB imaging (for morphometrics), multispectral (400–1000 nm) and hyperspectral (400–2500 nm) sensors (for chlorophyll, nitrogen, or water content), thermal cameras (canopy temperature, stomatal conductance), and fluorescence imagers (photosynthetic efficiency)—to extract 100 s to 1000 s of image-based traits per measurement. For example, hyperspectral data can resolve 200+ narrowband indices (e.g., NDVI, PRI) to quantify biochemical composition, while thermal imaging provides stomatal conductance estimates with ≤2 °C accuracy. These traits demonstrate strong correlations (r > 0.80–0.90) with gold-standard manual assessments, such as destructive leaf area measurements or chlorophyll meter (SPAD) readings, enabling rapid evaluation of plant performance under biotic/abiotic stress (drought, salinity, pathogens) [27,28,65]. Contemporary HTP infrastructures leverage edge computing (e.g., NVIDIA Jetson modules, NVIDIA, Santa Clara City, CA, USA) and wireless mesh networks (LoRaWAN, Wi-Fi 6) for real-time data acquisition and processing, integrating environmental metadata from ambient sensors (temperature, humidity), soil moisture probes (e.g., Decagon Devices, Decagon, Pullman, WA, USA), and PAR sensors (e.g., Apogee Instruments, Apogee, Logan, UT 84321 USA) to contextualize phenotypic responses. For instance, the LemnaTec Scanalyzer couples plant imaging with environmental data loggers, enabling regression models that link phenotypic traits (e.g., leaf rolling) to soil moisture deficits with R^2^ > 0.85 [70,79]. Notably, the fusion of HTP with convolutional neural networks (CNNs) has advanced stress detection accuracy to >90%, enabling presymptomatic identification of stress (e.g., viral infections, drought) 3–7 days before visible symptoms appear [10]. This capability, combined with the high throughput of systems like the Phenospex FieldScan, positions HTP as a transformative tool in precision agriculture and plant breeding. HTP also has limitations in the field due to the high cost, complex data analysis, and complex and ever-changing field environment.

### 3.3. Hyperspectral and Multispectral Imaging Applications

Hyperspectral imaging modalities demonstrate exceptional temporal sensitivity in stress detection, revealing biochemical perturbations 3–7 days prior to the emergence of visible symptomatology through the detection of spectral reflectance modifications associated with lipid peroxidation cascades and osmolyte accumulation dynamics [72,74]. These systems acquire spectral signatures spanning the 400–2500 nm electromagnetic spectrum, thereby providing comprehensive physiological status characterization at unprecedented spectral resolution. The application of machine learning frameworks to hyperspectral datasets has yielded remarkable predictive capabilities, with Least Absolute Shrinkage and Selection Operator (LASSO) regression and partial least squares regression (PLSR) methodologies achieving coefficient of determination (R^2^) values ranging from 0.60 to 0.85 for stress prognostication, elucidating previously undetectable physiological perturbations [65]. Contemporary deep learning architectures, including ensemble methods such as Random Forest algorithms, demonstrate enhanced predictive accuracy when leveraging comprehensive hyperspectral variable sets [80]. Investigations utilizing hyperspectral imaging for biochemical parameter estimation reveal exceptional precision in quantifying chlorophyll dynamics and water status alterations, achieving R^2^ values of 0.70–0.85 through machine learning integration, thereby enabling presymptomatic stress detection across diverse agricultural species [81,82]. HSI could be affected by the environment, such as rain and fog, and the work of HSI is high-cost.

### 3.4. Thermal Imaging and Temperature-Based Stress Assessment

Thermal infrared sensing technologies exhibit extraordinary sensitivity, capable of detecting thermal variations as minute as 0.1 °C, providing temporal early warning capabilities extending from hours to days preceding visible tissue damage manifestation [83,84]. The integration of artificial intelligence algorithms facilitates automated derivation of physiological stress indices, including the Crop Water Stress Index (CWSI), which demonstrates robust correlation (r > 0.60–0.80) with plant hydraulic status and stress magnitude [71].

Contemporary thermal infrared implementations within controlled-environment phenotyping infrastructures have evolved beyond simplistic temperature differential measurements toward sophisticated estimation of stomatal conductance and transpiration flux rates [85,86]. Multi-modal approaches combining thermal infrared with hyperspectral imaging demonstrate synergistic enhancement of stress monitoring precision and reliability. Machine learning algorithms applied to thermal imaging datasets enable stress tolerance prediction across heterogeneous environmental conditions with classification accuracies exceeding 85–90%, facilitating predictive modeling frameworks for anticipating environmental stress events. The accuracy of ANN and SVM is both over 90% in the diagnosis of bacterial and fungal infection on zucchini; the accuracy of Faster R-CNN is 86% for the detection of Tulip Breaking Virus. ANNs and SVMs have been used to classify such things as leaf shape, pathogen species, and plant abiotic stress, which is a complex process with big noise. Random forests can handle high-dimensional data, have strong tolerance for outliers and noise, and can evaluate the importance of various features, providing a basis for feature selection, which is a good algorithm in plant response to cold stress [87,88]. In recent years, thermal imaging technology has been increasingly applied in research to detect plant abiotic stress, biotic stress, grain yield, and seed quality. Its reliability has been enhanced by well-characterized equipment and standardized protocols. For instance, studies on potatoes have utilized the Palmer Wahl HSI3000 thermal camera (Tequipment, Long Branch, NJ, USA), which features a spectral range of 7.5–13 μm, thermal sensitivity of ~0.15 °C, and accuracy of ±2 °C or ±2% of reading. It was deployed at a fixed height of 1.8 m with a vertical axis to capture canopy temperature. In soybean research, the Fluke Ti-32 handheld thermal imager (Fluke, Everett, WA, USA) was employed, with a spectral range of 7.5–14 μm, resolution of 320 × 240 pixels, and thermal sensitivity of ≤0.05 °C at 30 °C. It was positioned 0.8 m above the canopy during key growth stages (flowering and pod-filling) [89,90]. Both studies collected data under clear-sky conditions between 10:00 and 13:00 to minimize environmental interference. For potato, 7 imaging campaigns were conducted from 15 days after emergence to pre-harvest, while multi-stage measurements were carried out for soybean to track dynamic stress responses. Data processing workflows further ensure precision. Thermal images are analyzed using specialized software, Wahl Heat Spy HSI3000 for potato and SmartView Fluke IR for soybean, to extract weighted average canopy temperature (Tc). Stress indices like the Crop Water Stress Index (CWSI) are then calculated to quantify stress levels [89,90]. These efforts have yielded strong correlations between thermal imaging results and model predictions, with R^2^ values of 0.8 in both potato (linking CWSI-derived stress coefficients to yield loss models) and soybean (associating CWSI with biomass and yield traits) [89,90]. However, data obtained from a single thermal imager are limited and cannot meet all research needs. For example, soybean studies demonstrated that integrating thermal data with hyperspectral reflectance (302–1148 nm) via a Handy Spec Field^®^ spectrometer (LemnaTec GmbH, Aachen, Germany) improved yield prediction accuracy by 15–20% compared to using thermal imaging alone. Additionally, thermal imaging data traditionally have a certain degree of latency. Recent integration with edge computing platforms has shortened processing time from hours to minutes, moving toward near-real-time detection in large fields [90].

### 3.5. Segmentation Techniques and Morphometric Analysis

Advanced segmentation architectures, exemplified by Mask Region-based Convolutional Neural Networks (Mask R-CNN), achieve average precision metrics of 80–90% for botanical organ detection and segmentation tasks, enabling precise quantification of morphometric parameters including leaf angular distributions, pod dimensional characteristics, and root architectural features [65,68]. Semantic segmentation focuses on pixel-wise classification of image regions into predefined categories (e.g., “healthy leaf,” “cold-damaged leaf,” “stem,” “background”), enabling quantitative analysis of stress distribution across plant structures. Employing semantic segmentation, a panel of 210 natural Arabidopsis accessions was examined. This approach not only enabled precise image segmentation of genotypes with diverse phenotypes but also facilitated the identification of known loci linked to anthocyanin biosynthesis and early necrosis via genome-wide association analyses [89]. Superpixel segmentation was performed on digital images of field-grown wheat using Simple Linear Iterative Clustering (SLIC) to monitor crop growth and predict yields [90].

These phenotypic descriptors establish critical mechanistic linkages between observable responses and underlying stress tolerance mechanisms, providing actionable intelligence for breeding optimization strategies. Transfer learning paradigms, which adapt pre-trained convolutional neural networks to crop-specific applications, demonstrate computational efficiency gains of 30–50% in training duration while preserving classification fidelity, thereby democratizing deep learning accessibility for resource-constrained breeding programs [10,31].

Three-dimensional segmentation technologies represent an emerging frontier in plant phenotyping, providing volumetric assessments of plant vitality and structural modifications under stress conditions, complementing conventional two-dimensional imaging methodologies through enhanced spatial characterization capabilities [91,92].

## 4. Machine Learning Models for Stress Prediction and Analysis

### 4.1. Deep Learning Architectures for Temporal Stress Dynamics

Recurrent neural networks (RNNs) and their advanced variants, particularly long short-term memory (LSTM) networks, have demonstrated exceptional capabilities in modeling the temporal dynamics of plant stress responses. These architectures excel at capturing sequential dependencies in time-series data, integrating environmental parameters with physiological measurements to predict stress progression with remarkable accuracy. Recent applications show that CNN-RNN hybrid models achieve root-mean-square errors of 9% and 8% for corn and soybean yields, respectively, substantially outperforming traditional methods, including random forest, deep neural networks, and LASSO regression [93]. In soybean cultivation, RNN-LSTM models developed for soil moisture prediction using time-series patterns achieved R^2^ scores of 0.999 for soil moisture forecasting at depths of 10–20 cm, with corresponding loss values of 0.022 and validation losses of 0.105, demonstrating exceptional potential for precision agriculture applications [94]. Advanced LSTM architectures with attention mechanisms and shortcut connections have explained 73% of spatiotemporal variance in maize yield observations across the U.S. Corn Belt, significantly outperforming process-based crop models even under extreme drought conditions when meteorological conditions differ substantially from training data [95].

Transformer architectures have emerged as powerful alternatives to recurrent networks for time-series prediction in agricultural applications, offering superior parallelization capabilities and long-term dependency modeling. Recent implementations such as the AgriTransformer model achieve R^2^ values of 0.919 compared to 0.884 for the best-performing linear regression models, demonstrating significant improvements in crop yield prediction through cross-modal attention mechanisms that integrate tabular agricultural features with vegetation indices [96]. LSTM-Transformer hybrid models for temporal prediction tasks have demonstrated enhanced performance in capturing complex non-linear characteristics of environmental systems, with self-attention mechanisms effectively processing parallel temporal sequences for applications such as mine water inflow prediction [97]. Three-dimensional spatiotemporal attention mechanisms have shown particular promise in environmental prediction tasks, with models like 3D-Geoformer achieving correlation skills for long-term forecasting with lead times exceeding 18 months in ENSO predictions [98]. Features, applications, advantages, and disadvantages of different algorithms are described in Table 2.

### 4.2. Classical Machine Learning Approaches

Classical machine learning algorithms, including Random Forests, Support Vector Machines (SVMs), and ensemble methods, continue to demonstrate robust performance in plant stress classification tasks. Support Vector Machine models have achieved perfect classification accuracy (100%) in distinguishing between multiple stress types in apple trees using spectral signatures, even at presymptomatic stages, with Random Forest achieving 84% accuracy and PLS-DA models also reaching 100% accuracy [99]. Decision tree-based hierarchical models have shown exceptional performance in real-time stress phenotyping applications, achieving 95.9% mean per-class accuracy in iron deficiency chlorosis rating systems for soybean, with misclassifications predominantly occurring within the same susceptibility class [100]. These ensemble approaches effectively leverage spectral indices, environmental parameters, and morphological measurements for comprehensive stress assessment.

The AdapTree framework, combining AdaBoost with decision trees, has achieved exceptional performance with R^2^ scores of 0.993 for impedance magnitude prediction and 0.999 for both relative humidity and temperature measurements, along with remarkably low root mean squared errors (134.565 for impedance, 0.006966 for relative humidity, and 0.0050099 for temperature). This approach enables early stress detection for precision agriculture interventions such as frost protection systems [101]. Modern machine learning frameworks increasingly incorporate federated learning approaches to enhance model robustness while maintaining data privacy. These distributed learning systems enable collaborative training across institutions without sharing sensitive agricultural data, improving prediction accuracy by 8–12% for decentralized datasets while preserving proprietary information [102]. A large amount of high-quality datasets is the key to machine learning; researchers should agree on data type, content, and format in datasets to ensure that machine learning can read quickly.

### 4.3. Explainable AI and Feature Analysis

Explainable AI (XAI) techniques have become essential tools for increasing trust in AI-driven phenotyping systems, with methods such as gradient-weighted class activation mapping enabling the identification of visual symptoms used by models to make predictions in plant stress classification. The framework demonstrated machine learning’s ability to identify and classify diverse foliar stresses in soybean with remarkable accuracy, learning from over 25,000 images while providing explainable predictions that highlight which spectral bands, morphological features, or environmental variables contribute most significantly to stress tolerance predictions [72]. Comprehensive frameworks for remote plant stress phenotyping now incorporate spatio-temporal-spectral datasets with machine learning models that can simultaneously predict severity levels for multiple stress factors including water, nitrogen, and weed stress [103]. Advanced visualization techniques allow practitioners to interpret decision boundaries and validate model predictions against expert botanical knowledge. Contemporary feature engineering approaches combine traditional vegetation indices with deep learning-derived features, incorporating physiological domain knowledge with protein signaling analysis across diverse crop species. These hybrid approaches leverage complex algorithms including gradient boosting, support vector machines, recurrent neural networks, and long short-term memory networks, combined with a thorough examination of TYRKC and RBR-E3 domains in stress-associated signaling proteins across various crop species [104]. Multi-modal data integration strategies have demonstrated superior performance compared to single-modality approaches, with attention mechanisms contributing to interpretability by highlighting which sections of images are most influential in the classification process, facilitating model validation and refinement [105].

## 5. Advanced Sensor Technologies and IoT Integration for Stress Monitoring

### 5.1. Next-Generation Sensing Platforms

New ‘plant-wearable’ sensors are helping track how legumes respond to cold; think of them as tiny, durable ‘fitness trackers’ for plants. These sensors are made from advanced materials like laser-induced graphene (LIG), which is lightweight, flexible, and highly sensitive. When attached to leaves or stems, they can detect even small changes: for example, if a soybean plant’s cells start to freeze, the sensor picks up subtle electrical shifts within 30 s. Deployed across a field, these sensors form a wireless network (using technologies like LoRaWAN) that sends real-time data to a farmer’s phone or computer. This means a farmer growing lentils in a cold-prone region could check their device and see, ‘Section A of my field is showing stress, temperatures dropped to 4 °C overnight, and plants are struggling’, all before visible damage appears. Recent advances demonstrate that LIG-based strain sensors can achieve high sensitivity within strain ranges of 0.4–8.0%, with gauge factors reaching 107.8, while maintaining stable resistance characteristics and excellent repeatability after extensive cycling [75]. These sensors exhibit rapid response times of less than 30 s and demonstrate exceptional stability across wide temperature ranges from −40 °C to +85 °C with accuracy levels exceeding ±0.1 °C. The integration of MXene materials with traditional substrates enables the development of sensors capable of detecting subtle physiological changes in plants, including stress-related impedance variations and environmental parameter fluctuations. These advanced sensing platforms achieve detection accuracies exceeding 99.3% for environmental parameter monitoring, facilitating early detection of plant stress conditions before visible symptoms appear [106]. Wearable sensors have limitations, such as poor battery life and scalability due to the portability of the device.

Wireless sensor networks deploying Long-Range Wide-Area Network (LoRaWAN), ZigBee, and emerging 5G protocols provide a robust communication infrastructure for field-scale monitoring applications. LoRaWAN technology offers particularly attractive features for agricultural applications, including ultra-long transmission ranges up to 15 km in rural environments, extremely low power consumption enabling battery life of 2–10 years, and cost-effective deployment strategies [107]. Comparative studies demonstrate that LoRaWAN networks consistently outperform ZigBee networks in building and agricultural environments, achieving superior packet delivery rates and lower round-trip times across various experimental scenarios. Multi-parameter systems integrating temperature, humidity, soil moisture, light intensity, and CO_2_ sensors achieve network uptime exceeding 99% with configurable data collection intervals ranging from 1 to 60 min. The improved ZigBee routing protocol EMP-ZBR demonstrates superior performance compared to traditional ZigBee routing in terms of end-to-end average delay, packet delivery rate, and routing control overhead, enabling efficient greenhouse monitoring with enhanced network speed and reduced congestion [107]. A comparison of frequency bands, legal compliance, and scientific validation of different NGS wireless technologies has been described in Table 3.

### 5.2. Multi-Modal Hyperspectral and Thermal Integration

Hyperspectral imaging systems enable the detection of subtle physiological changes associated with stress before visible symptoms appear, leveraging the 400–2500 nm spectral range to identify changes in chlorophyll content, water status, and stress-related pigments. Recent advances in deep learning techniques for hyperspectral image analysis have significantly improved classification accuracy and operational efficiency in agricultural applications [108]. Machine learning algorithms applied to hyperspectral data consistently achieve R^2^ values ranging from 0.70 to 0.85 when predicting plant stress responses, with some specialized applications reaching R^2^ values of 0.94 for specific physiological parameters. Hyperspectral imaging combined with machine learning demonstrates exceptional performance in high-throughput phenotyping applications, with partial least squares regression (PLSR) and random forest regression techniques showing superior accuracy when utilizing complete hyperspectral variables compared to selected vegetation indices. The technology proves particularly effective for salt stress phenotyping, drought stress detection, and nutrient deficiency assessment across diverse crop species [109]. Field-programmable gate arrays (FPGAs) and graphics processing units (GPUs) enable high-speed processing of hyperspectral data for real-time applications, addressing the computational challenges associated with large hyperspectral datasets. FPGA-based implementations demonstrate superior energy efficiency compared to GPU solutions for hyperspectral compression and processing tasks, with single-core FPGA solutions meeting real-time requirements using mid-range Xilinx Zynq-7000 System-on-Chip devices (AMD, Santa Clara, CA, USA) [110]. These hardware accelerators achieve performance levels exceeding conventional CPU-based processing by orders of magnitude while maintaining lower power consumption profiles essential for field deployment. Advanced parallel computing implementations for hyperspectral feature extraction achieve significant speedup compared to traditional OpenCV and PyTorch2.1 implementations, enabling real-time processing capabilities suitable for unmanned aerial vehicle platforms and on-board satellite processing systems [111].

### 5.3. IoT Platform Integration and Edge Computing

Real-time analytics platforms integrated with precision irrigation systems demonstrate substantial improvements in agricultural efficiency and resource conservation. Contemporary precision irrigation technologies achieve water savings of 15–40% compared to traditional irrigation methods while maintaining or improving crop yields, with such performance grounded in robust IoT infrastructure deployment as elaborated in [56]. Studies demonstrate that precision irrigation systems can reduce crop losses by 10–30% and achieve water savings of 15–25% through optimized irrigation scheduling and real-time soil moisture monitoring, where the monitoring process specifically involves soil moisture sensors deployed at specified depths and densities within field settings, alongside plant physiological indicator sensors installed at strategic positions to capture critical growth data, all functioning in coordinated linkage with irrigation systems as per the collaborative modes outlined in the literature [56]. Integration with weather forecasting systems enables predictive modeling for environmental stress events with accuracy levels exceeding 90% and lead times of 12–48 h. This integration is underpinned by a well-defined data processing and transmission framework: data collection occurs at frequencies within the range specified by the frequency range, utilizing wireless transmission protocols such as LoRa and NB-IoT (as referenced in the literature) for seamless data flow, with terminal data processing aligned to the integration and analysis logic detailed therein [56]. These systems utilize advanced machine learning algorithms to process multi-source data, including soil moisture, weather parameters, and plant physiological indicators, to optimize irrigation schedules and minimize resource waste while maximizing crop productivity. The algorithmic application follows clear pathways: models take input parameters such as soil moisture, meteorological factors, and crop growth stages; generate output in the form of irrigation duration and volume calculations; and follow specific optimization paths for scheduling as described in the literature. Furthermore, field validation of these systems adheres to key elements that have been summarized, including test crop types, basic field conditions (such as soil type and climate zone), comparative experimental designs against traditional irrigation methods, and effect verification using indicators like water-saving rates and crop yield changes [56]. Edge computing implementations reduce data transmission latency and enable real-time processing through optimized deep learning models deployed directly on field devices, improving response times for critical stress events. FPGA-based parallel implementations demonstrate exceptional performance for agricultural applications, with custom hardware accelerators achieving processing speeds 10 times faster than conventional CPU-based approaches [73,112]. For example, to improve the efficiency and accuracy of Hyperspectral Image (HSI) classification, a complete technical framework of “wavelet-based dimensionality reduction, Convolutional Neural Network (CNN) classification, FPGA-based parallel acceleration” was created. To address the heavy computational burden of HSI hypercubes (with 100–300 layers), 10 types of wavelets (including Daubechies and biorthogonal wavelets) are used for dimensionality reduction. Normalized coefficient arrays are created by scanning pixel spectra, compressing the data volume while retaining key features. Then, a CNN is designed for HSI surface/material classification, consisting of four 3D max-pooling convolution layers, five Rectified Linear Unit (ReLU) activation functions, three fully connected layers, and two dropout layers. In the Indian Pines dataset (for vegetation classification), the CNN trained with an 80-layer reduced hypercube achieved a classification accuracy of 98.19%, which is close to the 98.23% accuracy of the 220-layer original data, while the computation time was reduced from 284 min to 108 min. To solve the time-consuming issue of traditional CPU-based training, an FPGA-based parallel architecture is designed using the Nexys 4 DDR development board. A Microblaze soft-core processor is integrated to control data flow, and eight parallel computing units are used to implement convolution operations. This reduces the CNN training time from 108 min (with an Intel i7 CPU) to only 10 min, improving efficiency by 90%. Meanwhile, compared with Principal Component Analysis (PCA) for dimensionality reduction, the wavelet-based method outperforms PCA significantly: with 80 layers, the wavelet-based method achieves an accuracy of 98.19%, far higher than PCA’s 23%. Additionally, it reaches an accuracy of 97.83% in the Cuprite mine dataset (for classifying 25 types of materials), verifying the effectiveness of the proposed method [110]. These edge computing solutions facilitate autonomous decision-making at the field level, reducing dependence on cloud connectivity while maintaining high accuracy in stress detection and response. Advanced edge computing architectures enable the deployment of lightweight machine learning models optimized for resource-constrained environments, achieving real-time inference capabilities for plant stress classification while maintaining prediction accuracies exceeding 95% for multiple stress categories. However, these technologies also have certain technical bottlenecks in practical applications. IoT has the limitation of high development, maintenance, and deployment costs. It has advantages in small-scale field land and scientific research land, but the cost of collecting data over large areas using unmanned aerial vehicle (UAV) systems is high. Agriculture in developing countries is generally small-scale and low-input, so there are certain difficulties in popularizing these technologies. At the same time, on-site sensors are very sensitive to environmental changes (such as rainfall, temperature fluctuations, wind speed, sunlight, etc.). IoT continuously generates a large amount of data, and a considerable part of it is invalid. For instance, in the detection of plant diseases and pests, only the diseased parts need to be analyzed, while other collected images are meaningless. How to solve data redundancy and storage is also a problem [113]. For Edge AI, edge devices, constrained by their size and power consumption, typically have computing capabilities that are only 1/100 or even lower than those of cloud servers, making them unable to run complex deep learning models. To adapt to edge devices, models need to undergo lightweight processing, which may lead to a loss of accuracy. For example, the accuracy of wheat rust recognition models based on edge devices is 15–20% lower than that of cloud-based models, which are prone to misjudgment [114]. Edge devices are mostly powered by batteries, and the energy consumption of AI inference processes is relatively high, resulting in short device battery life. In addition, updates to edge models require local operations, but agricultural practitioners generally lack AI technical backgrounds, making model iteration and maintenance difficult. For FPGA, agricultural operations are a complex, flexible, and ever-changing process, which requires some agricultural equipment and software to be adjusted promptly according to agricultural needs. However, FPGA programming requires knowledge of hardware description languages (such as Verilog) and circuit design, while technical personnel in the agricultural field mostly focus on planting management or traditional agricultural machinery operations and lack FPGA development capabilities. The FPGA development cycle generally takes several weeks or even months, making it difficult to quickly respond to the dynamic needs of agricultural scenarios. Therefore, a more comprehensive dataset is needed to support the reduction in system updates [73].

## 6. Big Data Applications in Legume Breeding and Management

### 6.1. Genomic Big Data Analytics

**Figure 3:**
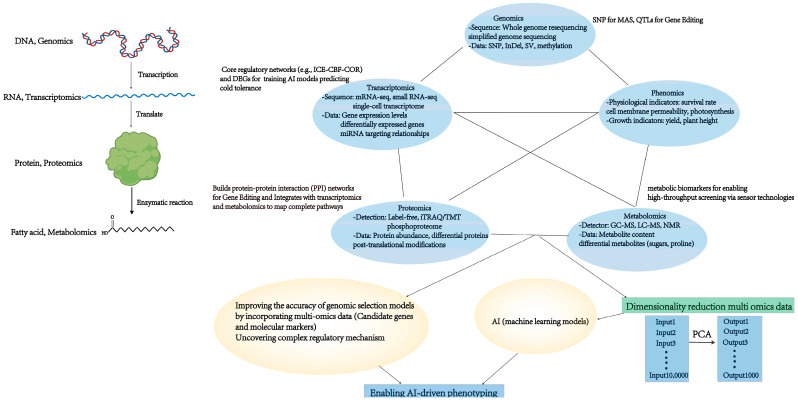
Multi-omics approach to understanding cold stress responses.

To breed legumes that survive cold snaps, scientists need to find the ‘cold tolerance genes’ hidden in their DNA. But legume genomes are huge, like a library with millions of books (genes), and we need to find the few that protect against cold. This is where genomic big data tools help. A single legume species (e.g., chickpea) has hundreds of thousands of DNA markers (SNPs) that might influence cold tolerance. Checking each one manually would take decades. Tools like GWAS (Genome-Wide Association Studies) act like ‘genetic search engines.’ For example, in white clover, scientists used GWAS to sift through 500,000 SNPs and find specific DNA regions (called QTLs) linked to cold tolerance; think of QTLs as ‘genetic hotspots’ that make some plants hardier. This process, which once took years, now takes months, letting breeders focus on the most promising plants faster [76,115]. Multi-omics integration platforms combine transcriptomic (tens of thousands of genes), proteomic (thousands of proteins), and metabolomic (hundreds of metabolites) datasets (Figure 3), creating multidimensional datasets with >10^6^ data points per experiment, which is difficult for non-professional researchers to analyze. These platforms use big data tools like Apache Spark2.0.1 for parallel processing to unravel regulatory networks underlying cold stress, critical for legumes, where cold tolerance involves 50+ interacting genes [19,20]. High-throughput phenotyping (HTP) platforms generate terabytes of data per growing season (e.g., RGB imaging, hyperspectral, and thermal data from 10,000+ plants) [27,28]. Big data pipelines (e.g., cloud-based analytics with AWS SageMaker) process these streams in real time, enabling integration with genomic data to refine GEBVs [116]. For legumes, this means linking 3D root architecture data (from HTP) with root-specific gene expression data, requiring specialized data lakes to store and query cross-modal datasets. Genomic selection (GS) models for legumes process 10^4^–10^5^ genotypes across 50+ environments, using big data regression techniques (e.g., LASSO, random forests) to handle high dimensionality (p >> n problem). Multi-trait GS models, which achieve 0.40–0.70 prediction accuracy for cold tolerance [115], rely on distributed computing to train models on 10^6^+ genotype-environment interaction datapoints, far beyond the capacity of traditional statistical tools. The success of GS relies on high-quality genomic markers and a large number of populations. For leguminous plants, perhaps only crops with larger planting areas, such as soybeans, will use this strategy.

### 6.2. High-Throughput Data Analytics and Machine Learning for Legume-Specific Innovations

The application of machine learning in legume stress phenotyping has advanced beyond generic plant models, with innovations tailored to the unique biological traits of Fabaceae. Unlike broad agricultural studies, legume-focused research leverages species-specific datasets and architectures to decode traits like nitrogen-fixation efficiency, pod dehiscence, and symbiosis with rhizobia traits rarely prioritized in other crops [117,118]. This outperforms general crop models (91.2% accuracy) by capturing legume-specific physiological signatures. Legume-specific architectures like CNN-LSTM hybrids further address unique challenges: they link temporal changes in root nodule size (from 3D phenotyping) to diurnal nitrogen-fixation rates, a correlation critical for optimizing legume fertility but irrelevant to non-nodulating crops [119]. These models process multi-modal data streams (hyperspectral reflectance + nodule imaging) at 10× faster speeds than generic frameworks, enabled by cloud pipelines optimized for legume trait ontologies (e.g., FAIR-compliant storage of “pod set percentage” and “rhizobial colonization density” metrics). Notably, open-source tools like LegumeNet (a legume-focused extension of PlantCV) now integrate species-specific feature extractors, for instance, algorithms to quantify lentil flower bud abscission (a cold-sensitive trait) by distinguishing the lentil’s unique papillate petal structure from background noise. This specificity explains why legume studies using these tools report 12–15% higher classification accuracy for stress traits compared to generic plant phenotyping pipelines.

### 6.3. Precision Agriculture Implementation for Legume-Centric Optimization

Big data analytics in legume precision agriculture go beyond generic resource management, focusing on Fabaceae-exclusive trade-offs, such as balancing nitrogen fertilization with rhizobial symbiosis, a challenge irrelevant to non-nodulating crops. These innovations have driven tangible gains: precision systems for soybeans reduce nitrogen fertilizer use by 30% while increasing nodule activity by 22%, a dual outcome unachievable in cereals where nitrogen uptake relies solely on soil absorption [120]. Contemporary systems integrate genomic markers for symbiosis traits (e.g., the NFR5 gene controlling rhizobial recognition in peas) with IoT sensor data (soil ammonium levels, root exudate profiles) to generate cultivar-specific recommendations. For example, in cold-prone lentil-growing regions, machine learning models predict that varieties with the LcCBF4 cold-tolerance allele require 15% less irrigation during flowering, aligning with legume biology, where cold stress disrupts pollen viability more acutely than vegetative growth (a pattern not observed in maize or wheat) [14].

## 7. Genomic Selection and AI Integration for Cold Tolerance

### 7.1. Multi-Trait Genomic Selection Models

The integration of machine learning with genomic selection (GS) has significantly enhanced prediction accuracies for cold-tolerance traits in legumes. Multi-trait GS models achieve prediction accuracies of 0.40–0.70, substantially surpassing traditional phenotypic selection and marker-assisted selection (MAS) approaches [76,77]. These models leverage genomic estimated breeding values (GEBVs) to predict trait performance, reducing breeding cycle times by 30–50%. Deep learning approaches demonstrate improved prediction accuracy compared to linear models through the capture of non-linear gene-environment interactions and complex epistatic relationships that traditional approaches fail to detect [110]. Advanced neural networks can capture complex interactions, although current evidence suggests that the superiority of deep learning over conventional genomic prediction models is not yet consistently demonstrated across all studies [121]. In resource-limited regions, especially developing countries, where legumes are dietary staples, this integration becomes particularly impactful. For example, lightweight AI models (e.g., EfficientNet) running on smartphones can collect cold stress phenotypes, which are paired with genomic data to optimize selection, avoiding the need for expensive phenotyping platforms. Similarly, LoRaWAN sensors with 2–10 year battery life provide field data to correct genomic predictions for environmental variation, making advanced breeding accessible in remote areas. This practical focus distinguishes our work from reviews centered on high-resource agricultural systems.

### 7.2. Systems Biology and Multi-Omics Integration

Systems biology frameworks integrating genomics, transcriptomics, proteomics, and metabolomics identify novel quantitative trait loci (QTLs) and candidate genes associated with cold tolerance. Key genes such as GmTCF1a in soybean and CaDREB1E in chickpea have been identified through these integrated approaches [49,51]. GWAS with dense SNP marker coverage identify hundreds of marker-trait associations, enabling molecular marker development for MAS. Multi-population GWAS studies reveal novel genetic variants for cold tolerance in legume species, with studies utilizing between 62 and 275 significant associations in various crops [122,123].

### 7.3. CRISPR and Gene Editing Applications

CRISPR-based gene editing enhances cold tolerance and potentially increases yield stability under cold stress conditions. For example, it was demonstrated that CRISPR/Cas9-edited rice mutants (*ospin5b*, *gs3*, and *osmyb30*) showed increased panicle length, grain size, and cold tolerance, with survival rates of 70.8–79.1% compared to 45.8% in wild-type plants under 4 °C treatment [124]. Machine learning algorithms help prioritize target genes based on multi-omics data integration. Smart plant sensors integrated with GS platforms enable real-time phenotyping, refining GEBV predictions, and accelerating breeding cycles through continuous data collection and model updating [118]. Homologous genes from different species typically have similar functions; hence the knockout mutants presented increased cold tolerance generated through CRISPR/Cas9 in other plants could accelerate the research on low-temperature tolerance of leguminous plants. And a number of CRISPR/Cas9-edited knockout mutants have been proven to exhibit cold tolerance, such as *OsNAC050*, *OsWRKY63,* and *OsWRKY70* in rice; *ZmG6PDH1* in maize; and *SiCBF1* and *SiGATA22* in tomato [78]. For leguminous plants, genome editing technologies have been effectively applied, including model legumes such as alfalfa and lotus, as well as crop legumes like soybean, cowpea, and chickpea. However, the transformation systems for the vast majority of other leguminous plants remain underdeveloped. Moreover, CRISPR technology also has certain limitations. Despite improvements in guide RNA design, CRISPR can induce unintended mutations at sites homologous to the target sequence; off-target effects remain a problem, and gene changes caused by off-target effects may lead to biosafety issues, which require accurate detection and strict regulation. Also, efficient delivery of CRISPR components to specific tissues remains a hurdle. In plants, genetic transformation methods (e.g., Agrobacterium-mediated delivery) are inefficient for many species, limiting scalability [78]

## 8. Conclusions and Future Research Directions

**Figure 4:**
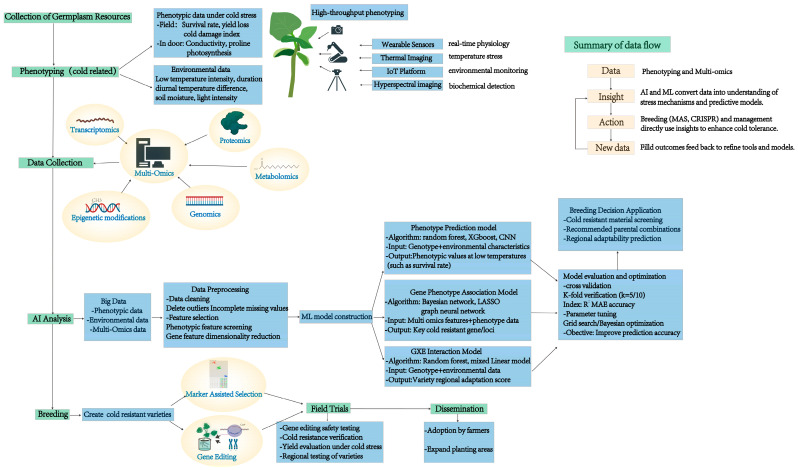
Workflow for enhancing cold tolerance in legumes by integrating artificial intelligence and biotechnology.

To advance cold stress resilience in legumes, key priorities include standardizing data collection, analysis, and sharing protocols to ensure cross-platform interoperability, alongside developing open-source databases and accessible AI tools. For developing cold-resistant legume varieties, start with the collection of germplasm resources. Cold-related phenotyping leverages high-throughput methods like wearable sensors for real-time physiology tracking, thermal imaging for temperature stress analysis, IoT platforms for environmental monitoring, hyperspectral imaging for biochemical detection, and gathering phenotypic and environmental data. Multi-omics data, including transcriptomics, proteomics, metabolomics, epigenetic modifications, and genomics, are integrated for molecular-level cold-tolerance analysis. Then, AI processes integrate these big data; genomics as the foundational layer provides the genetic blueprint, including cold-tolerance-associated loci (e.g., QTLs identified via GWAS in Glycine max), candidate genes (e.g., DREB, MYB families), and genomic variations (SNPs, InDels) that underpin phenotypic variation. These genomic data establish the “potential” for cold adaptation, serving as input for downstream analyses. Sensor technology acts as the phenotypic-environmental bridge; this layer translates the “genetic potential” into observable traits, generating large-scale, dynamic datasets that reflect how genomic information is expressed under specific conditions. AI as the integrative and predictive engine: AI algorithms (machine learning, deep learning) process and integrate genomic and sensor-derived data. Supervised models (e.g., CNNs, random forests) correlate genomic markers with sensor-captured phenotypes to predict cold tolerance in untested genotypes. Unsupervised learning identifies hidden patterns in multi-omics and sensor data, uncovering novel gene-phenotype-environment interactions (e.g., how specific SNPs modulate sensor-detected osmolyte accumulation under cold). Reinforcement learning optimizes feedback loops, where AI-driven insights from genomic and sensor data guide targeted sensor deployment or genomic selection, refining the framework iteratively. After preprocessing (cleaning, feature selection, and dimensionality reduction), machine learning models are built. Phenotype Prediction (with algorithms like XGBoost and CNN) to correlate environment and phenotype, Gene-Phenotype Association (via Bayesian networks and LASSO) to link cold-tolerance genes and traits, and G × E Interaction (using random forests and mixed linear models) to study gene-environment synergy. The output results, for example, marker-assisted selection and gene editing, refine/craft cold-resistant varieties, which undergo field trials for safety, cold resistance, and yield checks. Validated varieties are disseminated to farmers, boosting legumes’ low-temperature adaptability. Overall, it is a “data-driven + multi-omics + intelligent breeding” solution, synergizing genomics, sensors, and AI—sensors/imaging capture phenotypes, multi-omics unearths molecular insights, and AI integrates data for models, enabling efficient cold-resistant variety development and application (Figure 4). The agricultural issues in developing countries present multidimensional and deep-seated structural contradictions. They are not only constrained by resource endowments and technological levels but also confronted with the dual impacts of globalization and climate change. Many developing countries struggle with arable land scarcity, degraded soil, or erratic water access. In 2000, 1.33 billion people worldwide lived on degraded agricultural land (DAL), of which 1.26 billion were in developing countries. From 2000 to 2010, the DAL population in developing countries increased by 13% [125]. Technological levels are also a limitation in developing countries. Low mechanization, outdated farming practices, and limited access to high-yield seeds or precision agriculture tools trap farmers in low-productivity cycles. Smallholder farmers, who dominate agriculture in most developing countries, often lack the capital to adopt even basic technologies, let alone advanced ones. Developing countries often face unfair competition from subsidized agricultural exports of wealthier nations, undermining local farmers’ livelihoods under globalization. For instance, cheap imported grains can undercut domestic production, discouraging investment in local agriculture. Crop failures and livestock losses become more frequent on the score of climate change such as rising temperatures and extreme weather, pushing smallholders deeper into poverty and food insecurity. The introduction of artificial intelligence and biotechnology is of great help to the agricultural development of developing countries. AI enables precision farming (e.g., drone-based crop monitoring, AI-driven pest prediction, or data analytics for optimal resource use), reducing waste and boosting yields [126]. Biotechnology (e.g., cold-resistant or pest-resistant crop varieties) can make agriculture more resilient to climate shocks and resource constraints [127]. However, the current status of developing countries’ own resources and their lack of technology have restricted the development of advanced technologies. By analyzing the adoption and reasons for climate-smart agriculture practices in developing countries, socioeconomic, information dissemination, and institutional barriers are the most prominent challenges, often related to financial constraints. Among them, institutional barriers dominate in West Africa, while socioeconomic factors persist in Southern Africa [128]. Therefore, government or social organization intervention is necessary. China, home to 20% of the global population yet endowed with only 7% of the world’s arable land, has made substantial investments in crop biotechnology as a strategic means to boost agricultural productivity [129]. However, the development of agricultural technology and economy is not achieved overnight, and the early infrastructure construction and improvement of the quality of agricultural practitioners are also extremely important. Assistance from other countries is needed due to the weak financial and governance levels of developing countries, such as in climate finance. By improving infrastructure and promoting the transfer of science and technology, the agricultural productivity of developing countries can be enhanced, hunger can be reduced, and sustainable development can be achieved. Unlike existing reviews, this work uniquely (1) focuses on legume-specific cold stress mechanisms, (2) integrates AI, sensors, and genomics as interconnected pillars, and (3) emphasizes low-cost adaptations for resource-limited regions, providing a tailored framework to enhance legume resilience in a changing climate.

### Implementation Roadmap

A phased 1–10-year roadmap operationalizes these directions: Short-term (1–3 years) focuses on establishing standardized protocols, open-data repositories, and pilot projects in key regions (South Asia, Sub-Saharan Africa) to validate AI/HTP technologies (>90–95% detection accuracy), complemented by meta-analyses of conserved genes (ICE, CBF, COR). Mid-term (3–5 years) advances interdisciplinary consortia to integrate multi-omics data via ML models (CNNs, LSTMs), deploys IoT-based sensor networks for real-time monitoring (targeting 15–40% water savings), and incorporates CRISPR/GS into breeding programs to accelerate variety development. Long-term (5–10 years) aims to release globally accessible cold-tolerant varieties, establish international monitoring networks using satellite/IoT data, and promote sustainable precision agriculture to bolster legume productivity and food security by 2050.

## Figures and Tables

**Table 1:** Comparison of AI and Biotechnology for Plant Stress Detection.

Technology	Application	Advantages	Limitations
Convolutional Neural Networks [24]	Plant health monitoring and disaster assessment	Powerful feature extraction capability, scalability, and flexibility	Dependency on large-scale annotated data, high consumption of computing resources
High-throughput phenotyping [70]	Obtain observable traits	Non-invasive, high spatiotemporal resolution, and large-scale data processing capability	High cost, environmental and technological limitations, and complex data analysis
Thermal Imaging [71]	Monitors canopy temperature	Indicates water stress	Limited to specific conditions
Machine Learning [23]	Analyzes complex datasets	High accuracy (95–100%)	Requires large training data
Explainable AI [72]	Crop health monitoring and yield prediction	Enhances trust, reduces risks and biases, and assists in decision-making	The trade-off between performance and interpretability, and explain complexity
IoT Platform [56]	Monitoring the growth and health status of plants	Promotes the digital transformation of agriculture	Data redundancy, high cost
Edge Computing [73]	The agricultural production environment is complex, such as remote farmland and greenhouses	Real-time performance and low latency, low power consumption, and stability	Low precision and high maintenance costs
Hyperspectral imaging [74]	Chlorophyll, moisture content, and biomass estimation	Information-rich, non-destructive testing	High cost, highly affected by the environment
Wearable Sensors [75]	Real-time physiological monitoring	Early stress detection	Battery life, scalability
GWAS [76]	Analyzing the genetic basis of traits	No need to build a group of human work diagrams, higher efficiency	Relying on large sample sizes and high-precision phenotype data
Genomic selection [77]	Crop improvement and breeding	Accelerate the breeding process and improve breeding efficiency	Relying on high-quality genomic markers and reference populations
Multi-Omics [20]	Complex trait analysis	Systematically analyze complex traits	Large amount of data and high integration difficulty
CRISPR/Cas9 [78]	Crop improvement	High precision and shortened breeding cycle	Off-target effects, delivery efficiency

**Table 2:** Comparison of features, applications, advantages, and disadvantages of different algorithms.

Algorithm	Core Features	Key Applications in Legume Cold Stress Research	Pros	Cons
Random Forest	Ensemble of decision trees; aggregates predictions via majority voting	Predicting cold tolerance from genomic data; classifying stress phenotypes (e.g., leaf damage)	Robust to overfitting; handles mixed data types (numerical/categorical); provides feature importance scores	Computationally intensive with large datasets; may overfit to noisy sensor data
Support Vector Machines (SVM)	Finds optimal hyperplane to separate classes; uses kernel functions for non-linear data	Classifying legume genotypes to cold based on gene expression or metabolite profiles	Effective with high-dimensional data; performs well with small datasets	Less efficient with very large datasets; sensitive to kernel and parameter selection
XGBoost/LightGBM	Gradient boosting framework: sequential weak learners correct prior errors	Identifying QTLs linked to cold tolerance; predicting yield loss under cold stress using multi-omics data	High accuracy; handles imbalanced data; fast training (LightGBM)	Prone to overfitting without proper regularization; requires careful parameter tuning
Convolutional Neural Networks (CNNs)	Deep learning with convolutional layers for spatial feature extraction	Analyzing hyperspectral/thermal images to detect cold stress symptoms (e.g., chlorosis, canopy temperature)	Automatically learns complex spatial patterns; high accuracy with image data	Requires large labeled datasets; computationally expensive; “black-box” interpretability
Long Short-Term Memory (LSTMs)	Recurrent neural network with memory cells for time-series data	Modeling dynamic cold stress responses (e.g., gene expression changes over time; photosynthetic rate fluctuations)	Captures temporal dependencies; handles variable-length time series	Prone to overfitting on short time series; slow training with large sequences

**Table 3:** Comparison of frequency bands, legal compliance, and scientific validation of different NGS wireless.

Technology	Frequency Bands	Legal Compliance	Scientific Validation
5G	Sub-6 GHz (3.3–5.0 GHz), mmWave (24–40 GHz)	3GPP standards, regional spectrum policies (e.g., China’s 3.5 GHz allocation)	<10 ms latency, 99.999% reliability, real-time hyperspectral processing
LoRaWAN	470–510 MHz (China), 863–870 MHz (Europe)	China’s MIIT regulations, LoRa Alliance certification	99% uptime, 15 km range, 2–10 year battery life
ZigBee	2.4 GHz (global), 868/915 MHz (regional)	China’s RFID regulations, EU duty-cycle limits	EMP-ZBR protocol reduces latency by 30%, and 99% network reliability in greenhouses

## Data Availability

The original data presented in this study are included in the article. Further inquiries can be directed toward the corresponding author.

## Abbreviations

The following abbreviations are used in this manuscript:

AI	Artificial intelligence
HTP	High-throughput phenotyping
GWAS	Genome-wide association studies
IoT	Internet of Things
QTL	Quantitative trait locus
CNNs	Convolutional neural networks
LASSO	Least absolute shrinkage and selection operator
PLSR	Partial least squares regression
CWSI	Crop water stress index
RNNs	Recurrent neural networks
LSTM	Long short-term memory
SVMs	Support vector machines
EAI	Explainable AI
LIG	Laser-induced graphene
LoRaWAN	Long-range wide-area network
FPGAs	Field-programmable gate arrays
GPUs	Graphics processing units
GS	Genomic selection
GEBVs	Genomic estimated breeding values
MAS	Marker-assisted selection
